# Renal Tubular Acidosis an Adverse Effect of PD-1 Inhibitor Immunotherapy

**DOI:** 10.1155/2018/8408015

**Published:** 2018-01-31

**Authors:** Sandy El Bitar, Chanudi Weerasinghe, Elie El-Charabaty, Marcel Odaimi

**Affiliations:** ^1^Department of Internal Medicine, Northwell Health Staten Island University Hospital, Staten Island, NY, USA; ^2^Department of Hematology and Oncology, Northwell Health Staten Island University Hospital, Staten Island, NY, USA; ^3^Department of Nephrology, Northwell Health Staten Island University Hospital, Staten Island, NY, USA

## Abstract

Immune checkpoint blockade therapy is gaining popularity among oncologists for treatment of solid and hematologic malignancies. The widespread use of these agents resulted in increasing incidence of renal immune-related adverse events. Reported renal toxicity described so far includes acute interstitial nephritis, minimal change disease, and immune complex glomerulonephritis. We report the case of a 79-year-old female with metastatic non-small cell lung cancer on anti-PD-1 therapy nivolumab. After the 4th administration of nivolumab, the treatment course was complicated with normal anion gap metabolic acidosis. Urine and blood studies were in favor of distal renal tubular acidosis (RTA). Following a negative workup for an underlying etiology, immunotherapy-induced RTA was suspected. Withholding of the offending agent and initiation of steroid therapy resulted in adequate response. The present report provides the first presentation of RTA as a renal immune-related adverse event secondary to nivolumab. Nephrologists and oncologists should be familiar with potentially life-threatening renal side effects induced by immune checkpoint inhibitors.

## 1. Introduction

Novel therapeutic agents targeting PD-1 signaling are increasing in popularity among oncologists. Pembrolizumab and nivolumab have been approved by the US Food and Drug Administration for treatment of several malignancies and are showing high rates of durable clinical responses [1]. However, due to their immunologic effects, there have been a number of reported toxicities termed as immune-related adverse events (irAEs), classified and graded by the National Cancer Institute clinical terminology criteria of adverse events (CTCAE).

Renal adverse events are uncommon, with the highest rate reported in a phase II lung cancer trial at 4% [2, 3]. Three different forms of renal irAE have been described so far: acute interstitial nephritis, minimal change disease, and immune complex glomerulonephritis [4–7]. All three forms manifest as acute kidney injury (AKI) and rise in serum creatinine.

In this report, we present a case of nivolumab-induced renal tubular acidosis successfully treated with steroids and sodium bicarbonate.

## 2. Case Presentation

A 79-year-old woman with past medical history of stage IV non-small cell lung cancer (NSCLC), heart failure with preserved ejection fraction, and dyslipidemia presented to the emergency department with generalized weakness and fatigue. Patient was initiated on nivolumab 3 months prior to presentation as a second line treatment following failure of chemotherapy with carboplatin and pemetrexed, confirmed by progressive disease on PET/CT scan. Home medications included rosuvastatin, docusate sodium, and low-dose furosemide. Patient received nivolumab 240 mg every 2 weeks. Following her fourth dose, she started complaining of worsening generalized fatigue and progressive weakness. Upon outpatient evaluation, her creatinine was found to be elevated at 2.9 mg/dl from a normal baseline. Nivolumab and furosemide were held, and patient received intravenous fluid hydration in the clinic. A renal sonogram was unremarkable. Repeat blood work few days later showed improved renal function. However, the patient's functional status declined over the next few days limiting her out of bed activity. She was sent to the emergency department for further workup.

On admission, vital signs were within normal limits. Physical exam was unremarkable except for trace lower extremity edema bilaterally.

Initial blood work showed a sodium level of 137 meq/L, potassium of 2.4 meq/L, chloride of 116 meq/L, bicarbonate of 11 meq/L, BUN of 23 mg/dL, and creatinine of 1.67 mg/dL. Arterial PH was acidotic at 7.21 with a CO_2_ of 27 suggestive of nonanion gap metabolic acidosis with adequate respiratory compensation.

Urine analysis revealed few white blood cells and red blood cells but no casts. Urine studies demonstrated a urine PH of 6.5 and a urine anion gap of 22. The fractional excretion of sodium (FeNa) was calculated at 0.5%.

The clinical picture was suggestive of prerenal AKI (FeNa < 1%) and renal tubular acidosis (RTA). Gentle hydration with sodium bicarbonate drip was started, and the patient was given potassium supplementation.

On further investigation, the patient had a negative autoimmune workup except for an ANA of 1 : 320. SPEP, UPEP, free light chains, and hepatitis serology were negative. Thyroid function tests were within normal range.

The alkaline urine PH in the setting of a significantly low serum bicarbonate level suggested a distal-type RTA. After ruling out common etiologies of RTA, nivolumab was considered as the likely culprit for a drug-induced RTA.

On day 2 of hospitalization, repeat blood work revealed mild increase in serum bicarbonate to 13 meq/L and improved serum creatinine to 1.39 mg/dl. In the context of a suspected drug-induced RTA secondary to nivolumab irAE, the patient was started on dexamethasone 4 mg every 8 hrs and her fluid rate was increased to target administration of 3 mmol/kg/day of bicarbonate.

On day 4 of hospitalization, the serum bicarbonate increased to 19 meq/L and serum creatinine was back to baseline. Patient was transitioned to oral sodium bicarbonate and prednisone. Her functional status improved significantly, and she was discharged on day 6 of hospitalization. Her discharge labs revealed a sodium of 142 meq/L, potassium of 3.3 meq/L, chloride of 112 meq/L, bicarbonate of 21 meq/L, and creatinine at 0.95 mg/dL.

The patient was discharged home on oral bicarbonate and a prednisone taper.

Repeat labs 1 week after discharge were stable. Follow-up with nephrology and hematology was set up. However, the patient returned to the hospital with acute hypoxic respiratory failure due to massive pulmonary embolism secondary to heparin-induced thrombocytopenia and expired.

## 3. Discussion

The incidence and nature of renal irAEs in oncology are increasing with the introduction of immunotherapy. Cytotoxic T-lymphocyte antigen 4 (CTLA-4) and programmed cell death protein 1 (PD-1) are two important immune checkpoint receptors expressed by T lymphocytes and targeted by the new therapies. CTLA-4 plays a critical role in the early immune response occurring in lymphoid tissues. As T-cell receptors (TCR) bind antigen/MHC on antigen-presenting cells (APC), the coinhibitory receptor CTLA-4 overexpressed in many cancers is activated, leading to the inhibition of memory T-cell function. Following the priming and activation of T-cell lymphocytes, the coinhibitory PD-1 receptor expressed by T-cells of peripheral tissue binds to their ligands on cancerous cells (PDL-1 and PDL-2). This association results in T-cell anergy and decrease in cytokine release, hence suppressing the antitumor response [8, 9]. Nivolumab is a new PD-1 antibody with antitumor activity. It binds to PD-1 receptor and blocks its inhibitory pathway, hence stimulating lymphocyte cells to target tumor cells. It is now being used for treatment of non-small lung cancer, metastatic melanoma, renal cell carcinoma, squamous cell carcinoma of the head and neck, and classical Hodgkin lymphoma, as first or second line treatment [1, 2]. Studies have shown response rates as high as 40% in patients with solid tumors [2, 3]. In advanced NSCLC, response rate can reach up to 20% [2]. Nevertheless, these high numbers happen at the expense of several adverse effects. On a physiological level, immune checkpoint inhibitory pathways suppress immune response against self-antigens. Targeting these regulators may enhance an inflammatory response of normal tissue, resulting in immune invasion and damage [9]. The most common toxicities included cutaneous, gastrointestinal, hepatic, endocrine, and renal side effects. Hofmann et al. conducted a retrospective chart review study for reported adverse events of pembrolizumab and nivolumab noting renal involvement in only 1-2% of reported irAEs [10].

Three different forms of renal AEs have been reported so far: acute tubulointerstitial nephritis (ATIN), immune complex GN, and minimal change disease [4–7]. Irrespective of the underlying mechanism of irAEs, most respond well to steroid therapy. Based on the data derived from case series and case reports, treatment with glucocorticoids resulted in an excellent overall prognosis [10].

In our patient, the AKI was believed to be prerenal, considering the calculated FeNa was below 1% and the adequate response to intravenous fluids. However, the worsening acidosis despite improved renal function was suggestive of RTA. Extensive workup for an underlying etiology was negative, suggesting a drug-induced RTA secondary to nivolumab. Despite the elevated titer of ANA, it remains nonspecific as the rest of the autoimmune panel was negative. The alkaline urine PH despite significantly low serum bicarbonate and the adequate response to relatively low alkali supplementation was more in favor of a distal RTA. The underlying mechanism for autoimmune distal RTA is secondary to distal tubule proton pump defect from immunologic injury or autoantibodies [11]. Bearing in mind the importance of PD-1 signaling in minimizing the T-cell-mediated renal inflammation [10], anti-PD-1 agents are likely to induce immunologic injuries similar to that seen in autoimmune diseases resulting in RTA.

Establishing a relation between a certain drug and a side effect can be challenging, especially when a patient is exposed to multiple nephrotoxic drugs. In our case, the patient received maintenance pemetrexed, an antifolate drug, for 8 cycles. During the treatment period and 3 months after discontinuation of pemetrexed, her kidney function remained intact. Moreover, the association in time between administering nivolumab and the development of RTA, followed by normalization after administration of corticosteroids, is in favor of nivolumab-induced RTA.

Increase in use of immune checkpoint inhibitors should warrant attention to adverse side effects, mainly renal complications. To our knowledge, our case is the first to describe nivolumab-induced renal AEs manifesting as RTA. Early detection and initiation of steroid therapy/bicarbonate replacement can result in favorable outcomes. Renal monitoring for patients on anti-PD-1 agents is vital for early management of irAEs and prevention of severe renal damage.
